# Using a Goal Theoretical Perspective to Reduce Negative and Promote Positive Spillover After a Bike-to-Work Campaign

**DOI:** 10.3389/fpsyg.2019.00433

**Published:** 2019-03-06

**Authors:** Bettina Höchli, Adrian Brügger, Roman Abegglen, Claude Messner

**Affiliations:** Department of Consumer Behavior, Institute of Marketing and Management, University of Bern, Bern, Switzerland

**Keywords:** goal hierarchy, goal pursuit, behavior change, long-term, spillover effect, intervention, longitudinal multilevel analysis

## Abstract

Behavioral change interventions often focus on a specific behavior over a limited time period; for example, a bike-to-work intervention that incentivizes cycling to work over 2 months. While such interventions can successfully initiate behavior, they run the risk of triggering negative spillover effects after completion: Reaching the end of an intervention could reduce the motivation to maintain the behavior; or an increase in the targeted behavior (e.g., cycling to work more often) could lead to negative spillover across behaviors (e.g., cycling less in leisure time). Using a goal theoretical perspective, we tested whether an intervention focusing on a specific behavior during a limited time period (a subordinate goal) triggers negative spillover, and whether superordinate goals and/or action steps reduce negative or promote positive spillover. We conducted an experimental field study (*N* = 1,269) in the context of a bike-to-work campaign with a longitudinal multilevel design. Participants across all four experimental conditions had the campaign goal of cycling to work for a maximum of 2 months (a subordinate goal). A quarter of the participants additionally generated superordinate goals, a quarter action steps and a quarter superordinate goals *and* action steps. The last quarter was a control condition which only set the subordinate campaign goal. Surprisingly, the intervention caused no negative and some positive spillover effects. Participants increased the frequency of cycling to work across all groups and the increase could be maintained up to 2 months after the campaign. An increase in cycling to work spilled over to an increase in cycling in leisure time and to an increase in eating fruits and vegetables. No spillover effects were found regarding exercising and eating sweets and snacks. Participants focusing additionally on a superordinate goal cycled to work more frequently at the end of the campaign than the control group. Contrary to our expectations, the maintenance of cycling to work over time and the positive spillover effects across behaviors did not differ due to the goal manipulation. These results reduce the concern that interventions focusing on a subordinate goal could trigger negative spillover effects and show the need for additional experimental field studies.

## Introduction

Policy makers around the world are increasingly interested in how people's behavior can be changed (Frederiks et al., 2016). While regulatory mechanisms have traditionally been used to change behavior, campaign designers today increasingly rely on knowledge from behavioral research to motivate voluntary behavioral changes (Dolan and Galizzi, 2014; Moore and Boldero, 2017). In the environmental context, for example, there are numerous programs and interventions to encourage people to use less energy, focus more on renewable energy sources, produce less waste or switch to public transport, to name but a few (Abrahamse et al., 2005; for a review, see Osbaldiston and Schott, 2012; Abrahamse and Steg, 2013).

In order to be effective, behavior change interventions usually require people to adapt their behavior repeatedly over a long period of time and across different behavioral domains (Tiefenbeck et al., 2013; Moore and Boldero, 2017). To illustrate, one cannot lead a healthy life by exercising, or skipping dessert a single time. Thus, interventions aimed at changing behavior in the long-term and across behavioral domains have to consider not only the initiation of a targeted behavior, but also the long-term maintenance of an intervention effect, as well as possible effects that the change in the targeted behavior can have on other related behaviors. These effects are referred to as “spillover effects.” These spillover effects are positive when a first behavior increases the likelihood of engaging in a second related behavior and are negative when they decrease the likelihood of engaging in a second related behavior (e.g., Poortinga et al., 2013; Truelove et al., 2014; Dolan and Galizzi, 2015; Nilsson et al., 2017). Spillover effects can occur over time (when conducting behavior X affects the probability of conducting behavior X later on); across socio-spatial contexts (when conducting behavior X in one context affects the probability of conducting behavior X in another context) or across behaviors (when conducting behavior X affects the probability of conducting behavior Y, either in the same or in a distinct behavioral domain) (Nilsson et al., 2017).

In the context of goal setting theory, interventions that focus on the pursuit of a single concrete goal that describes *what* a person is trying to achieve in the short run (i.e., subordinate goals) have proven to be successful in initiating behavioral change. The motivational benefit of focusing on subordinate goals has been widely researched and documented (Abrahamse et al., 2005; Locke and Latham, 2013). However, if their effect is considered in the context of broad, long-term challenges that include possible spillover effects, it is unclear whether pursuing subordinate goals is still the most effective way to change behavior. Subordinate goals should not be used as a panacea for changing behavior within the design of interventions (Ordóñez et al., 2009). Potential negative spillover effects of subordinate goals are increasingly discussed; for example, interventions that focus on a subordinate goal are constrained in time and often focus specifically on the intervention period. Thus, they run the risk that people stop pursuing the goal as soon as the intervention has finished (Jeffery et al., 2000; Geller, 2002; Lally and Gardner, 2013). This can limit or even reverse possible intervention effects. We argue that when addressing broad, long-term challenges that require repeated behavior in the long-term and across different domains, superordinate goals fulfill a crucial role in motivating behavior, and a combination of both subordinate and superordinate is most effective (Höchli et al., 2018).

Using an experimental field study with a longitudinal multilevel design, the objective of this paper is to test whether (1) an intervention focusing on a subordinate goal gives rise to negative spillover effects over time and across behaviors, and whether (2) adding a superordinate goal can reduce negative and foster positive spillover effects over time and across behaviors. In order to better contextualize the results, a combination of a subordinate goal plus a concrete action step and a combination of all three—a subordinate goal, a superordinate goal and action steps—was tested.

## Using a Goal Theoretical Perspective to Reduce Negative and Promote Positive Spillover

In recent years, policy makers have started to consider how to address behavioral spillover in their campaign strategies (Lanzini and Thøgersen, 2014; Moore and Boldero, 2017). However, it is difficult to draw unequivocal conclusions about the design of interventions from previous research on spillover effects. Existing research has reported both positive spillover effects that foster the intended intervention effect (e.g., Whitmarsh and O'Neill, 2010; Thøgersen and Noblet, 2012; Willis and Schor, 2012) but also negative spillover that could nullify or even reverse the intended intervention effect (e.g., Sorrell, 2007; Barr et al., 2010). To date, no general consensus exists about when and why positive or negative spillover effects occur (Truelove et al., 2014).

These inconsistent and contradictory theories and results show the need for a deeper understanding of why positive and negative spillover effects occur and what conditions increase or decrease their likelihood (Whitmarsh and O'Neill, 2010; Truelove et al., 2014).

We take a goal theoretical perspective to explain why negative spillover effects occur and to offer a strategy for how negative spillover effects can be reduced and positive spillover effects can be promoted.

### Goal Hierarchy

When aiming to change behavior, the importance of planning and the usefulness of goals has been established (Carver and Scheier, 2001; Locke and Latham, 2013). Goals can differ in various characteristics, which can influence subsequent motivation and performance. To understand when positive and negative spillover effects occur, one characteristic of a goal is particularly relevant: the level of abstraction (Fujita and MacGregor, 2012). Concrete subordinate goals describe an action in detail: they convey exactly *what* action has to be done. As subordinate goals are constrained in time, and goal progress and achievement are easy to determine (e.g., Bandura, 1997), they can provide immediate incentives for performance and thus boost motivation. Abstract superordinate goals refer to idealized conceptualizations of one's self, one's relationships, or the society one is part of, and are closely linked with values. Superordinate goals constitute the reasons or motives for goal striving and convey *why* an action is performed. They are, by definition, more vague than subordinate goals but may better represent people's ultimate wishes and aspirations (e.g., Carver and Scheier, 2001), and promote vision and guidance (Locke and Latham, 2013).

Goals at different levels of abstraction are interconnected: Superordinate goals (e.g., living a healthy life) determine subordinate goals (e.g., lose 10 pounds) which in turn give rise to more concrete goals, such as action steps, that describe *how* to behave in a specific situation (e.g., run for 30 min as soon as one gets home from work on Tuesdays). Goals at different levels of abstraction can be seen as hierarchically ordered, with superordinate goals at the top and concrete goals at the bottom (e.g., Carver and Scheier, 2001).

### A Goal Theoretical Perspective and Negative Spillover

Focusing on subordinate goals has been shown to boost motivation and facilitate goal achievement. However, achieving a goal is not always an advantage. Achieving a goal can be negative because people stop working toward a goal when they perceive it to be completed (e.g., resting on laurels, Amir and Ariely, 2008; post-fulfillment inhibition, Förster et al., 2005; Zeigarnik effect, Zeigarnik, 1927). When pursuing a goal, the discrepancy between the status quo and the desired end-state results in an aversive and unpleasant tension (e.g., Carver and Scheier, 2001). In order to avoid this negative tension, people are motivated to decrease the discrepancy by acting in a goal-consistent way. Thus, the discrepancy encourages people to decrease the gap between their current state and their goal. Crucially, this also implies that once a goal is achieved, the discrepancy and the motivational impetus following from it will disappear. Goal achievement signals to people that they have done what is necessary and that they can stop pursuing that particular goal.

This tendency to relax one's efforts is unproblematic and even helpful if people really have achieved the goal they aspire to. However, many goals require continued effort over long periods of time. In addition, a goal is often only one of many steps that contribute to what is one's ultimate aspiration (i.e. their superordinate goal). Thus, achieving a subordinate goal (e.g., losing 10 pounds) will increase the tendency to relax efforts and may deter people from pursuing and achieving what they really want (e.g., living healthy life) and thus give rise to negative spillover over time. These arguments, which combine a goal theoretical perspective with negative spillover over time, are largely consistent with two other approaches explaining negative spillover effects: moral licensing and single-action bias (e.g., Truelove et al., 2014; Nilsson et al., 2017). *Moral licensing* occurs when a person who initially behaves in a moral way later on shows immoral, unethical or otherwise problematic behaviors (Mazar and Zhong, 2010; Merritt et al., 2010; Mullen and Monin, 2016). After doing good, a person thinks that she has done “enough” and allows herself to engage in less-moral behavior, believing she can balance out the prior moral and the latter less-moral behavior. *Single-action bias* occurs when a first action is perceived as a big step toward tackling a challenge or solving a problem, when in reality it was only a small step. As an illustration, a person who has insulated their house feels that this one action reduces climate change and therefore no longer considers it necessary to take further steps to prevent climate change (Hansen et al., 2004; Girod and De Haan, 2009).

Designing a campaign around subordinate goals could hinder positive and give rise to negative spillover effects not only over time but also across socio-spatial contexts and across behavioral domains. Subordinate goals motivate behavior as they focus attention on the goal-relevant behavior, which is crucial for goal pursuit (Locke and Latham, 2002). However, this focus can be too narrow, as when people overlook other important tasks that serve the pursuit of the goal in a broader sense (Ordóñez et al., 2009). For example, a person might focus on the goal of buying ecologically produced food for environmental reasons, without realizing that flying to Bali for the holidays contradicts her first behavior. Designing a campaign with a narrow focus on a subordinate goal could thus undermine positive spillover effects and foster negative spillover effects—especially across behaviors that are not similar, for example across socio-spatial contexts or across different behavioral domains.

Taken together, interventions that focus on a specific behavior over a limited time period—that is, behavior that focuses on a subordinate goal—may be prone to negative spillover effects both over time and across behaviors.

### A Goal Theoretical Perspective and Positive Spillover

One approach that might hinder negative spillover and foster positive spillover over time as well as across different behaviors is to design campaigns with a stronger focus on superordinate goals.

Superordinate goals can promote positive spillover effects over time as they often entail a long time span or do not have a clear end-state. In this case, achieving a subordinate goal or completing a campaign only signals partial fulfillment and the discrepancy between the status quo and the desired end-state is sustained. Because of this sustained discrepancy, people will not feel that they have “done enough,” which should motivate them to carry out further goal-consistent activities (Fishbach et al., 2006). This argument overlaps with several consistency theories that explain positive spillover effects, such as the foot-in-the-door effect (Freedman and Fraser, 1966) or the cognitive dissonance theory (Festinger, 1962; for a review on consistency theories, see Gawronski and Strack, 2012). These theories suggest that a first behavior activates a positive self-image or social identity and people infer feelings of distressing dissonance when acting inconsistently (Festinger, 1962). As a person tries to avoid this dissonance, the likelihood of performing a subsequent behavior that is consistent with the activated identity or concept increases (Truelove et al., 2014).

Furthermore, superordinate goals may foster positive behavioral spillover across socio-spatial contexts and across domains, as they interconnect several behaviors. When focusing on a superordinate goal, it becomes apparent that there are several means for pursuit (Kruglanski et al., 2002). For example, the goal of living a healthy life can be pursued by eating healthily, exercising regularly, and getting enough sleep. While these three distinct behaviors do not appear to be related in isolation, their interconnection becomes apparent when focusing on the common superordinate goal (Dolan and Galizzi, 2015). When a person focuses on a superordinate goal, engaging in a first goal-consistent action only signals partial completion, thereby motivating further actions. These further actions are not bound to the same or very similar repeated behavior, but can entail several distinct actions connected to the superordinate goal. For example, in order to progress toward a goal of “living a healthy life,” one could eat less convenience food, join a sports group, meditate, and get regular health checks. This also implies that, as long as the discrepancy between the status quo and the superordinate goal is sustained, a person will not engage in negative spillover behavior across other related contexts or behavioral domains, as the harmful effect of engaging in a behavior that contradicts the pursuit of the superordinate goal will be apparent.

Taken together, we argue that goals at all levels of abstraction have distinct advantages for the promotion of goal pursuit and work best when combined. Subordinate goals help to promote the initiation of a specific action, but they run the risk of triggering negative spillover effects. Superordinate goals are shown to be less motivating in initiating a behavior, but may be helpful to maintain a behavior over time as well as to foster positive spillover effects across other behaviors and domains. Thus, superordinate goals may help forestall negative spillover effects after reaching a first subordinate goal.

### The Present Study

To complement existing research on spillover effects, this study focuses on the spillover effects of an existing behavior change intervention (a bike-to-work campaign in Switzerland) over time and across behaviors in different socio-spatial contexts (cycling to work and cycling in leisure time) and in different domains (exercising, eating) in an experimental field setting. By taking part in the existing bike-to-work campaign, all participants pursued a subordinate goal defining *what* had to be achieved (i.e., cycling to work on at least half of the working days during the intervention period). We investigate whether the bike-to-work campaign, which focuses on a specific behavior over a limited period of time, triggers negative spillover effects over time (research question 1) and whether the campaign triggers negative spillover effects across behavior (research question 2). Based on the assumption that superordinate goals sustain discrepancy between the status quo and the desired state and that superordinate goals highlight the relationship between distinct behaviors, for both research questions we analyze whether adding a superordinate goal can reduce negative spillover and foster positive spillover over time and across behaviors.

In addition to a condition that combined the subordinate bike-to-work goal (*what*) with a superordinate goal (*why*), we also investigated a condition that combined the bike-to-work goal with concrete action steps that must be completed in order to achieve the bike-to-work goal (*how*). Focusing on *how* to achieve a goal has proven to be particularly helpful in the successful pursuit of goals when initiating a new behavior (see action phase model, Heckhausen and Gollwitzer, 1987) and when facing unfamiliar, complex situations (see control theory, Carver and Scheier, 1982; or action identification theory, Vallacher and Wegner, 1987). The advantage of action steps in goal pursuit is further reflected in the research on implementation intentions, which concentrates on *how* to achieve a goal and specifies in detail *when* and *where* this action will take place. In this way, implementation intentions link an intended action to a specific situation. Implementation intentions are shown to have a medium to large effect on promoting the initiation of an intended behavior (Gollwitzer and Sheeran, 2006) and are also helpful in maintaining a new behavior over time (Holland et al., 2006). Additionally, an experimental condition that references the empirically-supported, positive influence of action steps on goal achievement enables a better contextualization of the results (Watkins, 2011).

A combination of all three goal formulations is also examined as the final group of the study; this combination includes a subordinate goal (*what* do I pursue?), a superordinate goal (*why* do I pursue it?), and action steps (*how* do I pursue it?). It thus investigates how combining goals at different hierarchical levels could reap the benefits of superordinate goals, subordinate goals and action steps while canceling out the disadvantages (Höchli et al., 2018).

To summarize, the present study tests the following research questions.

Research question 1a: Does the effect of the bike-to-work campaign on cycling to work disappear at the end of the campaign and trigger negative spillover over time?

Research question 1b: Does formulating a superordinate goal and/or action steps in addition to the subordinate goal lead to a longer maintenance of the intervention effect on cycling to work, and therefore reduce negative and foster positive spillover effects over time?

Research question 2a: Does the effect of the bike-to-work campaign on cycling to work trigger negative spillover across behaviors?

Research question 2b: Does formulating a superordinate goal and/or action steps in addition to the subordinate goal reduce negative and foster positive spillover effects across behaviors?

## Methods

### Participants

Participants were recruited via official emails from the bike-to-work organization in Switzerland that were sent to all participants in the bike-to-work campaign. As an incentive, participants who completed the study were entered in a prize draw for 5 wellness weekends each worth CHF 800. The registration questionnaire was started by 1,842 people; of these participants, 309 did not complete the registration questionnaire, meaning that they could not be contacted and were excluded from the sample. Of the 1,533 participants who registered, 1,377 began the starting questionnaire, and out of these, 1,285 finished it and underwent the manipulation, thus meeting the minimal criteria to participate in this study. Within this sample, participants who changed their email address during the study and could no longer be uniquely identified were excluded from the study. Participants who were unable to provide meaningful answers regarding their cycling behavior (i.e., those who were injured or on holiday when they had to complete one of the questionnaires) were excluded from the corresponding questionnaire but remained in the sample for the remaining questionnaires. In addition, the study excluded responses regarding eating behaviors when the responses indicated that a person was consuming over 60 portions of fruit and vegetables per week (the total number of fruit and vegetable portions per week is determined by multiplying the number of days per week during which fruit or vegetables were eaten and the number of portions per day). Values above the mean at baseline plus six standard deviations, i.e., 60 portions per week, may indicate that those individuals have already indicated the number of portions per week rather than per day and were thus treated as inaccurate disclosures). But these participants were kept in the sample for the remaining questionnaires. Our final sample included 1,269 participants (746 women, 523 men, M_age_ = 38.57 years, SD_age_ = 10.89 years).

### Design

Participants were randomly assigned to one of four conditions of a between-subjects design with repeated assessment of the outcome variable (e.g., frequency of cycling to work) within 7 months starting at the end of the bike-to-work campaign. By taking part in the bike-to-work campaign, all participants committed to pursue the goal of cycling to work on at least half of the working days for a maximum of 2 months. The control condition focused solely on this subordinate goal. In addition to the subordinate goal, the first intervention condition was asked to think about *why* they wanted to bike to work and, on this basis, formulate a superordinate goal (*superordinate* condition); a second intervention condition was asked to think about *how* to meet the target of the bike-to-work campaign and, on this basis, formulate concrete action steps (*action step* condition); and a third intervention condition was asked to formulate action steps as well as a superordinate goal (*combined goal hierarchy* condition). As outcome variables, we measured the frequency of cycling to work (spillover effect over time), the frequency of cycling during leisure time (spillover effect across socio-spatial contexts), the frequency of exercising, and the frequency of eating healthy and unhealthy foods (spillover across behavioral domains) at the end of the campaign and up to 7 months afterwards.

### Procedure

Data were collected by the research team via seven online questionnaires: A registration questionnaire (1), an initial questionnaire at the start of the campaign (2), an end questionnaire at the end of the campaign (3), and three follow-up questionnaires (4–6). Additionally, a final follow-up questionnaire (7) was sent 7 months after the end of the campaign, in the winter, to all participants who had agreed to be contacted again. During the campaign, participants received a reminder message approximately every 2 weeks.

**Figure 1:**
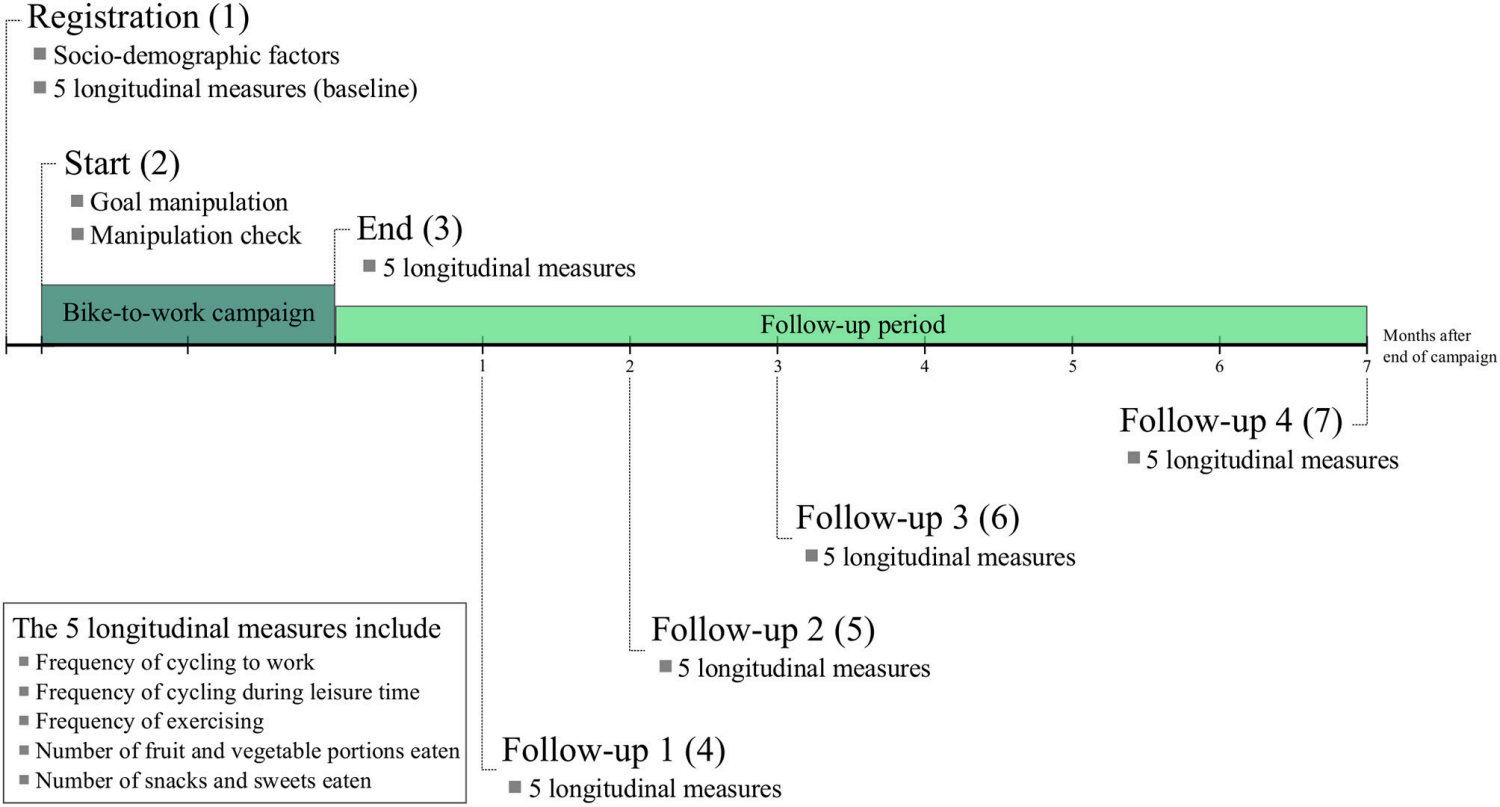
Variables measured at the separate time points.

The registration questionnaire was sent 1 week before the start of the campaign. Consent for participating in the research was attained by asking participants to continue only if they had read the provided instructions, agreed to them, and were willing to participate in our study. To establish a baseline, we asked participants how frequently they cycled to work and during their leisure time, as well as about their exercising and eating behaviors. Furthermore, participants answered socio-demographic questions. The starting questionnaire was sent out the day that the campaign started. In the starting questionnaire, participants completed the goal manipulation and a manipulation check. To make sure that participants did not forget the details of the experimental condition they were assigned to, they received reminder messages approximately every 2 weeks during the campaign. On the last day of the campaign, participants received the end questionnaire. It assessed their frequency of cycling to work, cycling in their leisure time, and exercising, and also assessed their eating behaviors. Participants answered the same questions 2, 3, and 7 months after the end of the campaign (see Figure 1). All study elements were designed in Qualtrics and distributed via email.

Various additional variables were assessed which are not topic of this article (e.g., whether participants interpreted their behavior as progress or commitment, or the level of self-efficacy), and thus will not be described in the material and will not be evaluated in this context.

### Measures and Materials

#### Goal Manipulation

The control condition (*N* = 327) focused only on the goal of the bike-to-work campaign: that is to cycle to work on at least half of the working days during the campaign.

The first intervention condition (superordinate goal, *N* = 316) was asked, in addition to the bike-to-work goal, to consider why they would like to pursue the bike-to-work campaign goal and write down their answer in their own words. Participants were then asked to address their answer and explain why it was important to them and again write down their answer. With these considerations in mind, participants were asked to consider which greater life goal the bike-to-work campaign and the desire to ride a bike more often is connected with, and to formulate a personal goal starting with “I want to be a person who…” (for a similar approach see laddering technique, e.g., Reynolds and Gutman, 1988).

The second intervention condition (action steps, *N* = 311) was asked, in addition to the bike-to-work goal, to write down three specific behaviors that will help them to achieve the bike-to-work campaign goal successfully. Participants were informed that ideally, these should be new behaviors that they have not yet implemented regularly and want to repeat. They were then asked to select the behavior that seemed to be the easiest and most effective to implement, and to formulate it as a personal goal. The third intervention group (combined goal hierarchy, *N* = 315) was asked, in addition to the bike-to-work goal, to formulate both action steps and a superordinate goal.

#### Manipulation Check

To measure the hierarchical level of abstraction of participants' goals, participants rated their goal on a 5-point scale using eight semantic differential items (adapted from Burrus, 2006, Cronbach's α =.78): from *central to life as a whole* (=1) to *side issue for life as a whole* (=5), from *complicated* to *simple*, from *long-term goal* to *short-term goal*, from *concerns life as a whole* to *concerns a specific aspect of life*, from *focusing on why something gets done* to *focusing on how something gets done*, from *influences overall path of life* to *influences minor detours in life*, from *is strongly linked to personal values* to *is detached from personal values*, and from *important* to *not important*. For the control condition, this rating refers to the subordinate goal of the bike-to-work campaign; for the superordinate and combined goal hierarchy conditions to their self-formulated superordinate goal and for the action step condition to their self-formulated action step.

#### Longitudinal Measures

Five variables were measured on six separate time points: as baseline measurement just before the start of the campaign (baseline measurement), at the end of the campaign (end measurement), and after 1, 2, 3, and 7 months after the end of the campaign (4 follow-up measurements). Figure 1 gives an overview of the variables measured at the separate time points.

Participants were asked on how many of the past 7 days they cycled to work, they cycled in their leisure time and they did strenuous and moderate physical activities. Furthermore, participants were asked on how many of the past 7 days they have eaten vegetables and fruits as well as sweets and snacks, and the number of portions of each they ate on average per day. To compute the total number of fruit and vegetable portions as well as snacks and sweets eaten, the number of days was multiplied by the average number of portions of the respective food.

## Results

The results are presented in three parts. First, we report several data quality checks. Second, we describe the spillover effects of the intervention over time, both for the sample as a whole and separately for the four experimental conditions (research question 1). Third, we describe the spillover effects of the intervention across behaviors, again both for the sample as a whole and separately for the four experimental conditions (research question 2).

### Data Quality Checks

#### Attrition Analysis

Among the participants who completed the start questionnaire, not all completed all five subsequent questionnaires (end questionnaire and four follow-up questionnaires, M = 4.09, SD = 1.344). To examine potential bias introduced by differential attrition between groups, we compared the number of completed questionnaires across groups but did not find any differences, [*F*_(3, 1265)_ = 0.69, *p* = 0.556]. That is, there is no reason to assume that the conditions had an effect on the motivation to participate.

#### Manipulation Check

To test whether the goal manipulation had the intended effect, we measured the self-reported hierarchical abstraction of participants' goals. A Kruskal–Wallis test showed differences among the four goal conditions, χ^2^(3) = 167.63, *p* < 0.001. Follow-up tests were conducted to evaluate pairwise differences among the four groups, controlling for Type I error across tests by using the Bonferroni approach. A Kruskal–Dunn test indicated that participants who formulated a superordinate goal (superordinate goal condition and combined goal hierarchy condition) assessed their goal as more abstract than did the control condition and the action step condition (see Table 1), which indicates a successful manipulation.

**Table 1:** Kruskal–Dunn comparisons of self-reported hierarchical abstractions of participants' goals.

				**Kruskal–Dunn comparisons (bonferroni)**		
**Group**	***n***	**Mean**	**SD**	**Combined goal hierarchy**	**Superordinate goal**	**Action steps**
Combined goal hierarchy	315	2.42	0.51			
Superordinate goal	316	2.42	0.56	1.000		
Action step	311	2.72	0.58	<0.001	<0.001	
Control	327	2.84	0.48	<0.001	<0.001	0.007

#### Randomization Check

To check whether randomization was successful, a one-way multivariate analysis of variance (MANOVA) with baseline measures of cycling to work, cycling in leisure time, intensive physical activity, moderate physical activity, eating fruit and vegetables, and eating snacks and sweets as the dependent variables and condition (control vs. action step vs. superordinate goal vs. combined goal hierarchy) as the independent variable was performed. The MANOVA did not reveal a significant multivariate effect, [*F*_(3, 1205)_ = 1.32, *p* = 0.180], and no significant univariate effects, indicating successful randomization (all *p* > 0.153).

### Effects of the Bike-to-Work Campaign Over Time

To answer our first research question, the spillover effects are analyzed over time; first in relation to the overall intervention effect (research question 1a) and then in relation to the four experimental goal manipulation conditions (research question 1b).

#### Overall Effect of the Campaign on Cycling to Work Over Time: More Rides to Work Until Two Months After the Campaign

Our data—that is, repeated measurements on individuals—had a hierarchical structure with measures nested within persons. Accordingly, we analyzed the data by applying a hierarchical linear modeling approach using the R-package lme4 (Bates et al., 2014). The first level of analysis was at the repeated-measures level (i.e., respondents reported longitudinal measures on cycling to work at the six measurement points at the within-person level). The second level of analysis was at the level of the individual respondent and captured changes in behavior between individuals.

In order to assess the overall effect of the campaign on cycling to work (research question 1a), we fitted a multivariate, multilevel model with random intercepts (for model specification see Supplementary Model 1). We examined the mean change of cycling to work at each of the five-measurement point compared to the baseline measure before the campaign and tested whether these means differed significantly. Results of this multivariate multilevel model are presented in Table 2.

**Table 2:** Application of a multivariate multilevel model for a within-subjects pre-/post-design with six fixed occasions.

	**Fixed**						**Random**	
**Predictor**	**Coef**.	***b***	**SE**	**df**	***t***	**95% CI**	**Coef**.	**SD**
**MODEL 1: CYCLING TO WORK**								
Intercept	β_00_	2.98	0.05	2589.45	54.87^***^	2.88 to 3.09	*r*_oi_	1.49
End	β_10_	0.88	0.05	5246.79	17.51^***^	0.79 to 0.98		
Follow-up 1	β_20_	0.35	0.05	5264.59	6.75^***^	0.25 to 0.45		
Follow-up 2	β_30_	0.27	0.05	5274.69	5.09^***^	0.16 to 0.37		
Follow-up 3	β_40_	0.09	0.05	5277.66	1.70	−0.01 to 0.19		
Follow-up 4 (winter)	β_50_	−0.65	0.06	5304.74	−11.14^***^	−0.76 to −0.54		

*Coef. = Coefficient in corresponding model equation; b = unstandardized regression coefficient; N_occasions_ = 6,459, N_persons_ = 1,269*.

* p < 0.05,

** p < 0.01,

*** *p < 0.001*.

**Figure 2:**
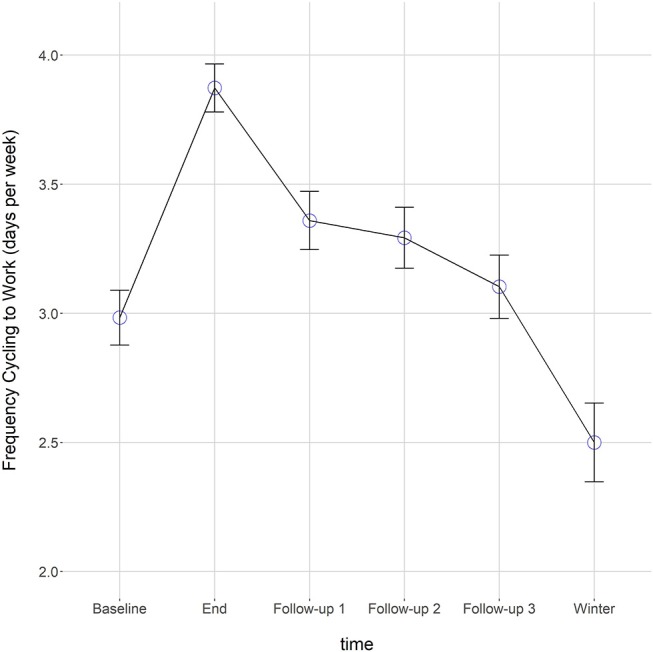
Mean frequency of cycling to work for all participants at six measurement points.

At the end of the campaign, participants cycled to work on average almost 1 more day (0.88) per week than they did before the campaign, *b* = 0.88, *t* = 17.51, *p* < 0.001. This positive effect, when compared to baseline, was still present (although to lesser extents) 1 month, *b* = 0.35, *t* = 6.75, *p* < 0.001 and 2 months after the end of the campaign, *b* = 0.27, *t* = 5.09, *p* < 0.001. Three months after the end of the campaign, the positive effect on cycling to work was no longer discernable as the frequency of cycling to work was similar to baseline measurement, *b* = 0.09, *t* = 1.70, *p* = 0.09. Seven months after the end of the campaign—which corresponded to the winter season in Switzerland—participants cycled to work less often than they did at baseline, *b* = −0.65, *t* = −11.14, *p* < 0.001. In short, participants cycled more frequently during and up to 2 months after the campaign. Three months after the campaign, however, they returned to the same frequency as before the campaign, and in winter the frequency dropped below baseline levels (see Figure 2).

#### Effect of the Goal Type Manipulation on Cycling to Work Over Time: Superordinate Goals Show Some Positive Effects

To assess how cycling to work will develop after the end of the campaign and answer research question 1b, model 1 was slightly adapted. On the first level of analysis (the repeated-measures level within an individual), we included five measures per participant starting with the measurement at the end of the campaign where time was set to zero. The baseline measurement of cycling to work was included as a covariate at the between-person level. Furthermore, to assess whether formulating a superordinate goal and/or action steps in addition to the subordinate goal leads to longer maintenance of the intervention effect on cycling to work, we included goal type as a second-level (between persons) predictor. On this basis, we fit a multilevel growth model with random intercepts and random slopes as justified by the data (for model specifications see Supplementary Model 2). Results are presented in Table 3.

**Table 3:** Application of a multilevel growth model examining the effect of goal type on cycling to work.

	**Fixed**						**Random**		
**Predictor**	**Coef**.	***b***	**SE**	**df**	***t***	**95% CI**	**Coef**.	**SD**	**Slopes < 0**
**MODEL 2: CYCLING TO WORK**									
Intercept	β_00_	3.61	0.07	1178.41	50.87^***^	3.47 to 3.74	*r*_oi_	0.96	
Baseline cycling to work (cgm)	β_01_	0.59	0.02	1203.68	35.53^***^	0.56 to 0.63			
Combined goal hierarchy	β_02_	0.14	0.10	1170.97	1.39	−0.05 to 0.34			
Superordinate goal	β_03_	0.21	0.10	1180.74	2.03^*^	0.01 to 0.41			
Action step	β_04_	0.06	0.10	1172.84	0.62	−0.13 to 0.26			
Time	β_10_	−0.21	0.02	930.26	−11.00^***^	−0.25 to −0.17	*r*_1i_	0.19	87.13%
Combined goal hierarchy: time	β_11_	−0.02	0.03	910.05	−0.64	−0.07 to 0.04			
Superordinate goal: time	β_12_	−0.01	0.03	920.15	−0.33	−0.06 to 0.04			
Action step: time	β_13_	0.03	0.03	926.24	1.05	−0.02 to 0.08			

*Coef. = coefficient in corresponding model equation; b = unstandardized regression coefficient; slopes < 0 = percentage of random slopes that were estimated to be negative (calculated on the basis of the assumption of normally distributed random slopes; see Hox et al., 2017); N_occasions_ = 5,190, N_persons_ = 1,269. The baseline measure of cycling to work was centered at the grand mean*.

* p < 0.05,

** p < 0.01,

*** *p < 0.001*.

The Intercept (β_00_) shows that at the end of the campaign the control group cycled to work on average 3.61 days per week. At the between-person level, the frequency of cycling to work before the campaign (β_01_, baseline) has a positive effect on cycling to work after the campaign across individuals, *b* = 0.59, *t* = 35.53, *p* < 0.001, indicating that people who cycled frequently before the start of the campaign were also more likely to cycle more frequently at the end of the campaign. The coefficients β_02−_β_04_ shows the effect of the goal manipulation on cycling to work at the end of the campaign. For the group with an additional superordinate goal, a positive change in mean at the end of the campaign compared to the control group was observed, indicating that the campaign had a stronger effect for participants with a superordinate goal compared to the control group, *b* = 0.21, *t* = 2.03, *p* = 0.020, Pseudo-*R*^2^ = 0.003^1^. No differences were observed between the combined goal hierarchy and the control condition or between the action steps and the control condition.

At the within-person level, time had a negative effect on cycling to work (β_10_), indicating that the frequency of people riding their bike to work is declining after the end of the campaign, *b* = −0.21, *t* = −11.00, *p* < 0.001, Pseudo-*R*^2^ = 0.25. This negative trend over time was observed for 87.13% of the sample (the percentage of individuals for whom the time slope was negative; see Hox et al., 2017). Thus, for the large majority of participants, the frequency of cycling to work decreased over time. This result is consistent with the results regarding the overall effect of the campaign: People maintained an increased level of cycling to work up to 2 months after the campaign. Three months after the intervention, the frequency of cycling to work did not differ from baseline, and 7 months after the campaign, during winter, a significant decrease compared to baseline was observed.

To test whether the goal manipulation had an effect on cycling to work over time—that is to see whether additionally formulating a superordinate goals and/or action steps could reduce or even dissolve this negative trend on cycling to work over time—the cross-level interaction between goal manipulation and time (β_11−_β_13_) is of interest. For the goal manipulation to be effective at fostering cycling to work in the long-run, we would expect β_11−_β_13_ to be significantly larger than zero. The cross-level effects of all three goal manipulations x time did not yield any significant effects. This indicates that complementing a subordinate goal with a superordinate goal and/or action steps did not lead to longer maintenance of the positive intervention effect, and thus did not mitigate the decrease of the target behavior over time.

### Effects of the Bike-to-Work Campaign Across Behaviors

To answer our second research question, the spillover effects are analyzed over across behaviors; first in relation to an increase in cycling to work (research question 2a), and then in relation to the goal manipulation (research question 2b).

#### Spillover Effects of the Campaign Across Socio-Spatial Contexts and Behavioral Domains: Partly Positive Effects From an Increase in Cycling to Work

The frequency of cycling to work increased on average across all participants as a result of the intervention. In the next step, to answer research question 2a (whether an increase in cycling to work could trigger negative spillover across behaviors), we investigated spillover effects from this change in cycling to work to cycling in leisure time, as well as across behavioral domains such as exercising and eating. We used a series of longitudinal multilevel models (Supplementary Models 3–6), to examine the effect of a change in cycling to work on the four possible spillover behaviors. The respective possible spillover behavior is the first-level outcome variable and cycling to work is the first-level predictor variable centered at the individuals mean; it is denoted by the suffix “cwc” (or “centered within clusters”; Enders and Tofighi, 2007). Additionally, we took the mean of all five measurements of cycling to work as a second-level predictor to control for the mean cycling frequency of each person. And finally, we included the baseline measure of cycling to work and the baseline measure of the respective possible spillover effect as a second-level predictor. All second-level predictors are denoted with the suffix “cgm” (or “centered at grand mean”; Enders and Tofighi, 2007). All models included random intercepts and random slopes as justified by the data (for model specifications, see Supplementary Models 3–6). Results of these multilevel models are presented in Table 4.

**Table 4:** Application of multilevel models examining the relation between cycling to work and four possible spillover behaviors.

	**Fixed**						**Random**		
**Predictor**	**Coef**.	***b***	**SE**	**df**	***t***	**95% CI**	**Coef**.	**SD**	**Slopes < 0**
**MODEL 3: LEISURE CYCLING**									
Intercept	β_00_	1.97	0.03	1196.60	63.31^***^	1.91 to 2.03	*r*_oi_	0.92	
Baseline leisure cycling (cgm)	β_01_	0.58	0.02	1172.72	32.33^***^	0.54 to 0.61			
Baseline cycling to work (cgm)	β_02_	−0.06	0.02	1230.04	−2.40^*^	−0.10 to −0.01			
Person mean cycling to work (cgm)	β_03_	0.28	0.03	1248.54	10.04^***^	0.23 to 0.34			
Cycling to work (cwc)	β_10_	0.17	0.02	625.99	10.40^***^	0.14 to 0.21	*r*_1i_	0.21	79.9%
Person mean cycling to work (cgm): cycling to work (cwc)	β_11_	0.03	0.01	955.53	2.31^*^	0.00 to 0.05			
**MODEL 4: EXERCISE**									
Intercept	β_00_	3.50	0.05	1187.40	75.62^***^	3.42 to 3.60	*r*_oi_	1.41	
Baseline exercise (cgm)	β_01_	0.54	0.02	1190.41	28.14^***^	0.50 to 0.58			
Baseline cycling to work (cgm)	β_02_	0.02	0.03	1227.99	0.48	−0.05 to 0.09			
Person mean cycling to work (cgm)	β_03_	0.05	0.04	1237.82	1.1	−0.04 to 0.13			
Cycling to work (cwc)	β_10_	0.04	0.02	511.78	1.82	−0.01 to 0.09	*r*_1i_	0.28	55.8%
Person mean cycling to work (cgm): cycling to work (cwc)	β_11_	0.08	0.02	795.05	4.62^***^	0.04 to 0.11			
**MODEL 5: FRUITS AND VEGETABLES**									
Intercept	β_00_	21.25	0.23	1151.52	90.89^***^	20.80 to 21.72	*r*_oi_	7.35	
Baseline fruits and vegetables (cgm)	β_01_	0.47	0.02	1152.88	27.76^***^	0.44 to 0.51			
Baseline cycling to work (cgm)	β_02_	−0.04	0.17	1185.93	−0.21	−0.40 to 0.33			
Person mean cycling to work (cgm)	β_03_	0.26	0.21	1195.22	1.23	−0.15 to 0.67			
Cycling to work (cwc)	β_10_	0.31	0.08	3731.46	3.99^***^	0.15 to 0.47	*r*_1i_	0.09	99.9%
Person mean cycling to work (cgm): cycling to work (cwc)	β_11_	−0.08	0.06	3741.39	−1.39	−0.20 to 0.03			
**MODEL 6: SNACKS AND SWEETS**									
Intercept	β_00_	6.63	0.1	1192.13	65.04^***^	6.43 to 6.84	*r*_oi_	3.05	
Baseline snacks and sweets (cgm)	β_01_	0.51	0.02	1178.04	31.26^***^	0.48 to 0.54			
Baseline cycling to work (cgm)	β_02_	0.11	0.08	1243.20	1.38	−0.04 to 0.26			
Person mean cycling to work (cgm)	β_03_	0.01	0.09	1248.65	0.16	−0.17 to 0.20			
Cycling to work (cwc)	β_10_	0.03	0.05	513.45	0.7	−0.06 to 0.12	*r*_1i_	0.39	53.3%
Person mean cycling to work (cgm): cycling to work (cwc)	β_11_	0.02	0.03	829.83	0.61	−0.04 to 0.08			

*Coef. = coefficient in corresponding model equation; b = unstandardized regression coefficient; slopes < 0 = percentage of random slopes that were estimated to be negative (calculated on the basis of the assumption of normally distributed random slopes; see Hox et al., 2017); N_occasions_ = 5,190, N_persons_ = 1,269. The baseline measure of cycling to work was centered at the grand mean*.

* p < 0.05,

** p < 0.01,

*** *p < 0.001*.

All baseline values of the behaviors that we tested for potential spillover effects had a positive effect on the respective potential spillover behavior in all four models (see Table 4). For example, participants who cycled more frequently in their leisure time before the campaign also cycled more frequently in their leisure time after the campaign. The baseline value of cycling to work only showed a small negative effect on cycling in leisure time, *b* = −0.06, *t* = −2.40, *p* = 0.017, Pseudo-*R*^2^ = 0.004.

At the between-person level, individual means of cycling to work predicted cycling in leisure time, *b* = 0.28, *t* = 10.04, *p* < 0.001, Pseudo-*R*^2^ = 0.10, indicating that people who on average cycle more to work also cycle more in their leisure time.

**Figure 3:**
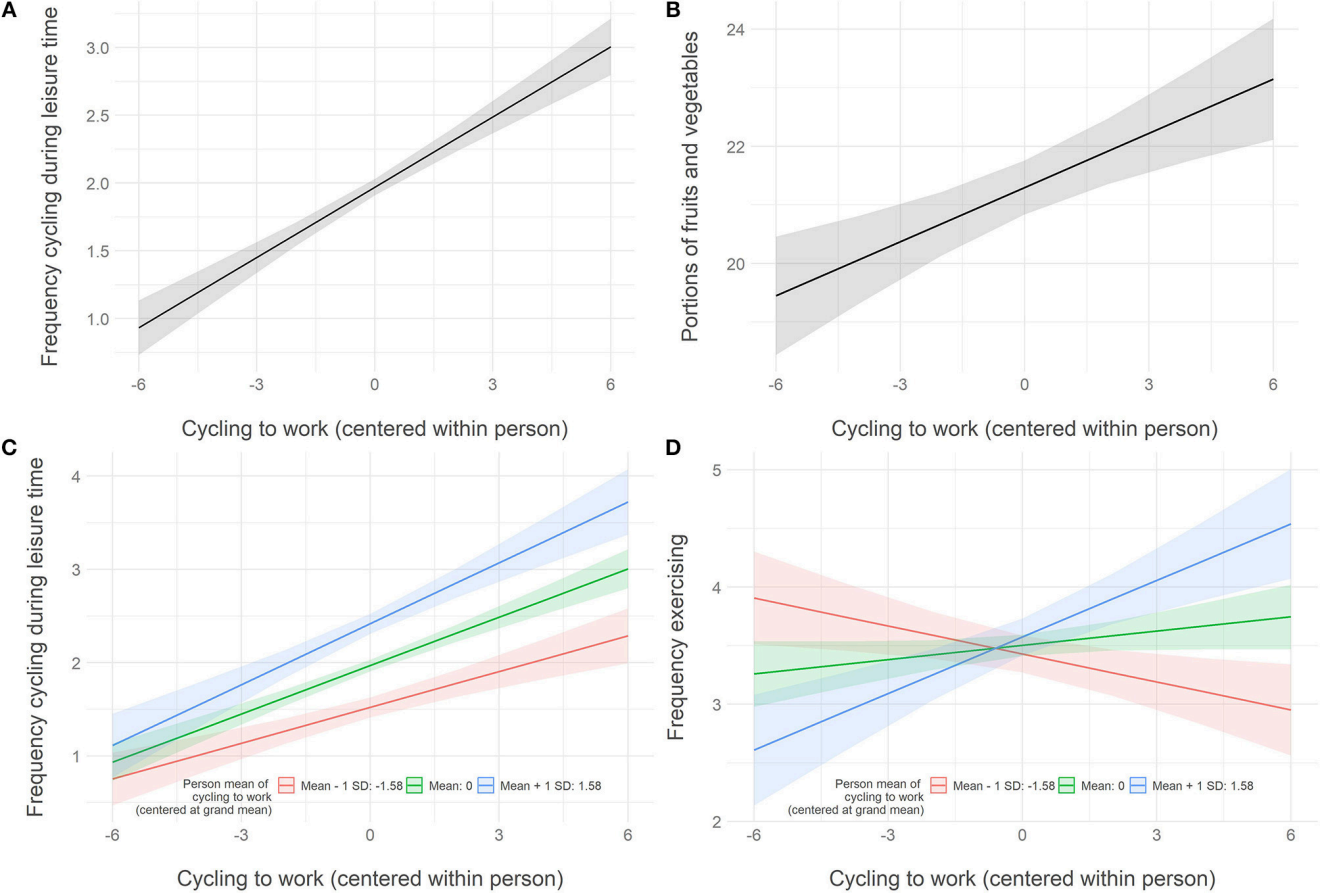
**(A,B)** respectively show the frequency of cycling during leisure time and portions of fruits and vegetables eaten as a function of the frequency of cycling to work (centered within persons). **(C,D)** show the relationship between the frequency of cycling to work and, respectively, the frequency of cycling during leisure time **(C)** and the frequency of exercise **(D)** for three different levels of person means of cycling to work.

To answer the research question whether an increase in cycling to work gives rise to spillover effects across behaviors, the within-person level is of importance. At the within-person level, cycling to work positively predicted cycling in leisure time, *b* = 0.17, *t* = 10.40, *p* < 0.001, Pseudo-*R*^2^ = 0.09, and eating fruits and vegetables, *b* = 0.31, *t* = 3.99, *p* < 0.001, Pseudo-*R*^2^ = 0.005 (see Figures 3A,B). No effect was found regarding exercising, and eating snacks and sweets.

The individual differences in cycling to work moderated the within-person slope for cycling to work regarding cycling in leisure time, *b* = 0.03, *t* = 2.31, *p* = 0.021, and exercising, *b* = 0.08, *t* = 4.62, *p* < 0.001. This indicates that participants with a higher level of individual means of cycling to work showed a larger positive spillover effect on cycling in leisure time and on exercising than participants with a lower level. In the case of exercise, even a change from a positive spillover for persons with a high person-mean to a negative spillover for persons with a low person-mean can be observed (see Figures 3C,D).

#### Spillover Effects of the Goal Type Manipulation Across Socio-Spatial Contexts and Across Behavioral Domains: No Effect of the Goal Manipulation

Although the goal manipulation did not have a consistent statistically significant impact on cycling to work, it is still possible that the goal manipulation affected other behaviors (Lanzini and Thøgersen, 2014). To answer research question 2b, whether goal manipulation can hinder negative and foster positive spillover effects across behavior, we tested whether there is a more positive change in cycling in leisure time, exercising and eating in the intervention groups than in the control group.

We repeated the statistical analyses in Table 3 with the exception of the respective possible spillover behavior replacing cycling to work as the dependent variable and the baseline of the respective possible spillover behavior replacing the baseline of cycling to work (see Supplementary Models 7–10). All models included random intercepts and random slopes as justified by the data. The results are presented in Table 5.

**Table 5:** Application of multilevel growth models examining the effect of goal type on several spillover behaviors.

	**Fixed**						**Random**		
**Predictor**	**Coef**.	***b***	**SE**	**df**	***t***	**95% CI**	**Coef**.	**SD**	**Slopes < 0**
**Model 7: LEISURE CYCLING**									
Intercept	β_00_	2.24	0.08	1195.92	29.79^***^	2.10 to 2.38	*r*_oi_	11.08	
Baseline cycling leisure (cgm)	β_01_	0.63	0.02	1174.62	35.42^***^	0.60 to 0.67			
Combined goal hierarchy	β_02_	0.02	0.11	1189.82	0.2	−0.18 to 0.23			
Superordinate goal	β_03_	0.02	0.11	1200.28	0.2	−0.18 to 0.24			
Action step	β_04_	0.00	0.11	1190.28	0.04	−0.19 to 0.21			
Time	β_10_	−0.14	0.02	963.20	−8.56^***^	−0.17 to −0.11	*r*_1i_	0.12	87.8%
Combined goal hierarchy: time	β_11_	0.00	0.02	944.15	−0.1	−0.05 to 0.04			
Superordinate goal: time	β_12_	0.02	0.02	955.05	0.98	−0.02 to 0.07			
Action step: time	β_13_	0.01	0.02	957.81	0.42	−0.03 to 0.06			
**MODEL 8: EXERCISE**									
Intercept	β_00_	3.63	0.1	1203.59	34.95^***^	3.42 to 3.85	*r*_oi_	1.50	
Baseline exercise (cgm)	β_01_	0.54	0.02	1190.65	28.06^***^	0.50 to 0.58			
Combined goal hierarchy	β_02_	−0.09	0.15	1199.31	−0.62	−0.37 to 0.20			
Superordinate goal	β_03_	−0.12	0.15	1208.39	−0.82	−0.42 to 0.18			
Action step	β_04_	0.16	0.15	1199.12	1.09	−0.12 to 0.47			
Time	β_10_	−0.04	0.02	943.45	−1.74	−0.08 to −0.01	*r*_1i_	0.15	60.3%
Combined goal hierarchy: time	β_11_	0.01	0.03	925.38	0.26	−0.05 to 0.07			
Superordinate goal: time	β_12_	−0.03	0.03	935.85	−0.81	−0.09 to 0.03			
Action step: time	β_13_	−0.04	0.03	938.55	−1.21	−0.10 to 0.02			
**MODEL 9: FRUITS AND VEGETABLES**									
Intercept	β_00_	21.81	0.51	1155.92	43.03^***^	20.75 to 22.89	*r*_oi_	7.74	
Baseline fruits and vegetables (cgm)	β_01_	0.48	0.02	1155.09	27.89^***^	0.44 to 0.51			
Combined goal hierarchy	β_02_	0.09	0.73	1149.31	0.13	−1.24 to 1.56			
Superordinate goal	β_03_	0.61	0.73	1155.91	0.84	−0.86 to 2.01			
Action step	β_04_	−0.51	0.73	1153.15	−0.70	−1.95 to 0.88			
Time	β_10_	−0.21	0.09	886.11	−2.30^*^	−0.38 to −0.02	*r*_1i_	0.63	62.9%
Combined goal hierarchy: time	β_11_	−0.03	0.13	858.59	−0.27	−0.29 to 0.21			
Superordinate goal: time	β_12_	−0.12	0.13	867.12	−0.94	−0.35 to 0.13			
Action step: time	β_13_	−0.17	0.13	874.78	−1.29	−0.43 to 0.11			
**MODEL 10: SNACKS AND SWEETS**									
Intercept	β_00_	3.07	0.26	1309.71	11.99^***^	2.55 to 3.58	*r*_oi_	3.26	
Baseline snacks and sweets (cgm)	β_01_	0.51	0.02	1175.19	31.10^***^	0.48 to 0.54			
Combined goal hierarchy	β_02_	−0.27	0.33	1197.31	−0.81	−0.92 to 0.41			
Superordinate goal	β_03_	0.06	0.33	1204.77	0.19	−0.61 to 0.70			
Action step	β_04_	−0.46	0.33	1201.48	−1.40	−1.13 to 0.22			
Time	β_10_	0.03	0.05	930.61	0.64	−0.06 to 0.13	*r*_1i_	0.33	46.6%
Combined goal hierarchy: time	β_11_	−0.13	0.07	907.91	−1.93	−0.27 to 0.00			
Superordinate goal: time	β_12_	−0.09	0.07	915.85	−1.33	−0.23 to 0.05			
Action step: time	β_13_	−0.02	0.07	921.19	−0.33	−0.16 to 0.11			

Coef. = coefficient in corresponding model equation; b = unstandardized regression coefficient; slopes < 0 = percentage of random slopes that were estimated to be negative (calculated on the basis of the assumption of normally distributed random slopes; see Hox, 2010, p. 19); N_occasions_ = 6345, N_persons_ = 1269. All baseline measurements as well as the person means of cycling to work were centered at the grand mean. p < 0.1,

* p < 0.05,

** p < 0.01,

*** *p < 0.001*.

The baseline of the respective behavior had, in all models, a positive effect on the respective behavior (see Table 5). At the between-person level, goal manipulation had no effect on the four spillover behaviors. At the within-person level, time had a negative effect regarding cycling in leisure time, *b* = −0.14, *t* = −8.56, *p* < 0.001, Pseudo-*R*^2^ = 0.13, and eating fruits and vegetables, *b* = −0.21, *t* = −2.30, *p* = 0.022, Pseudo-*R*^2^ = 0.07.

To test research question 2b, whether goal manipulation can hinder negative (and foster positive) spillover effects across behaviors, the cross-level interaction between goal manipulation and time (β_11−_β_13_) is of importance. For the goal manipulation to be effective at fostering the four spillover effects in the long-run, we would expect β_11−_β_13_ to be significantly larger than zero. None of the three goal manipulations x time interactions yielded any significant effects, indicating that the goal manipulation did not affect the spillover behaviors over time.

## Discussion

Many individual and societal challenges require people to change their behavior over the long-term and across several behaviors. Thus, intervention designers have to take into account not only a specific, time-bound targeted behavior but also possible spillover effects of this targeted behavior, across time and across behaviors. However, no general consensus exists about the direction and size of possible spillover effects, nor about which factors can promote positive spillovers and reduce negative spillovers (Truelove et al., 2014). Furthermore, studies testing spillover effects experimentally in the field are still scarce and there is a need for more experimental research (Lanzini and Thøgersen, 2014). To contribute to this, based on a goal theoretical perspective, we tested whether an intervention focusing on a specific behavior over a limited period of time (i.e., a subordinate goal) gives rise to negative spillover effects over time and across behaviors, and whether the formulation of a superordinate goal and/or action steps can hinder negative and foster positive spillover effects.

The campaign was successful in various aspects: Irrespective of the goal conditions, participants cycled to work more often at the end of the campaign than they did before the campaign. The increase in the cycling frequency was maintained up to 2 months after the campaign and thus the risk that the intervention effect will disappear immediately after the end of the intervention was not confirmed. While the results indicate that focusing on a superordinate goal increased the intervention effect measured at the end of the campaign, no effect of the goal manipulation was observed regarding the maintenance of the intervention effect over time. An increase in cycling to work spilled over across socio-spatial contexts to an increase in cycling in leisure time, and across behavioral domains to an increase in eating fruits and vegetables, which does not confirm the risk of negative spillover across behaviors. However, counter to our expectations, the goal manipulation did not yield any effect on the direction or size of the spillover effects across behaviors.

### Spillover Effects in the Field

Embedding the present study in an existing large-scale campaign allows for an experimental design that enables the investigation of spillover effects in the field. Thus, the results of this study provide several insights on spillover effects across time and across behaviors in field settings. To start with, the overall increase in cycling to work compared to baseline for up to 2 months after the end of the campaign somewhat reduces the concern that the effect of a time-limited intervention will only last as long as the intervention itself (Jeffery et al., 2000; Geller, 2002; Lally and Gardner, 2013). Nevertheless, 2 months is a short period, and the decline in the intervention effect back to the initial level 3 months after the end of the campaign indicates that the participants did not change their behavior sustainably in the long-run (Lally and Gardner, 2013).

Furthermore, the evidence emerging from this study does not support the concern of negative spillover effect in field studies that could potentially nullify or even reverse the intervention effect on the targeted behavior, but corroborates earlier findings suggesting that behavior can, under certain circumstances, positively spill over from one behavior to other related behaviors (e.g., Lanzini and Thøgersen, 2014; Chatelain et al., 2018). The observed spillover effects are not very strong, although small effect sizes are not unusual in the context of spillover (see Blanken et al., 2015). However, the results show no consistent positive spillover effect across all observed behaviors, suggesting that the occurrence of spillover effects depends on certain attributes of the observed behaviors. There are at least two relevant attributes in this respect: similarity between and cost of the behaviors. Spillover effects—negative and positive—are more likely to occur between similar behaviors (Truelove et al., 2014). Similarity may be with respect to the behavioral domain but also to the cost and effort or frequency of performance, to the symbolic meaning of the behavior, or to how the behavior is performed (Lanzini and Thøgersen, 2014). This is consistent with our finding that an increase in cycling to work positively spills over to an increase in cycling in leisure time. Furthermore, earlier findings suggest that individuals are more likely to adopt new behaviors that are not costly, and spillover is more likely to impact low-cost than high-cost behavior, where cost in the broad sense can refer to any kind of expenditure (e.g., money, time, physical strength, attention) (Lanzini and Thøgersen, 2014). This line of research may explain why, in the present study, an increase in cycling to work positively spilled over to healthy eating but not to unhealthy eating and exercising. It can be hypothesized that the costliness and effort of the specific spillover accounts for the observed effects: Performing an additional workout requires more time and physical effort than eating an additional apple. As spillover is more likely to impact low-cost than high-cost behavior, an increase in cycling to work is more likely to spillover to eating more fruits and vegetables, which requires relatively low effort, and less likely to spillover to exercising, which requires relatively high effort. Furthermore, a decrease in eating sweets and snacks can be seen as resisting a temptation. Temptations offer an immediate outcome which exerts a strong motivational pull (Fishbach et al., 2003) and thus often stand in conflict with goals that are higher in importance but whose outcomes are less salient and further away (Cavallo and Fitzsimons, 2012). Resisting temptation is difficult and requires high effort and willpower (Gollwitzer et al., 2010). If eating sweets and snacks is considered a temptation, observing no spillover effect is consistent with earlier results suggesting that spillover is less likely to impact high effort behaviors.

Finally, the results show the relevant role of moderating variables in the occurrence of spillover effects—namely, the average frequency of conducting the targeted behavior. While the positive spillover effect of *cycling to work* to *cycling in leisure time* was greater for people who, on average, cycled more frequently to work, the spillover effect on exercising was even reversed depending on the average frequency of cycling to work. Alternatively, the spillover effect was positive for those who, on average, cycled more frequently to work, and it was negative for those who cycled less often to work. This gives us the first indication of the possible risk of compensatory behavior: for people who conduct a target behavior infrequently, an increase in the target behavior could lead to a reduction in the associated behavior (for a similar reasoning, see Brügger and Höchli, submitted).

### The Role of a Goal Theoretical Perspective in Spillover Effects

While some results indicate that focusing on a superordinate goal as well as a subordinate goal reinforces the positive intervention effect, there was no consistent positive impact of the goal manipulation—both superordinate goals and/or action steps—on spillover effects.

The lack of effect of action steps on cycling to work does not support previous results. The effect of action steps has been widely studied and shows positive effects on goal pursuit across various domains (see for example research on implementation intentions, Gollwitzer and Sheeran, 2006). While the focus of this technique is mainly on initiating behaviors (e.g., Gollwitzer, 1999; Brandstätter et al., 2001), there are also some studies that highlight the advantage of implementation intentions for maintaining behavior over time, especially in combination with further self-regulatory measures such as mental contrasting (e.g., Stadler et al., 2010; Oettingen, 2012; Duckworth et al., 2013). However, our results show no effect of formulating action steps on cycling to work during the bike-to-work campaign as well as up to 7 months after the campaign. We can speculate that many people participating in the bike-to-work campaign already cycled before the campaign started and some of them may have already developed the habit of cycling to work. Some evidence for this explanation comes from research on implementation intentions: Implementation intentions are shown to have a strong effect on adopting a new behavior (Gollwitzer and Sheeran, 2006) or breaking old unwanted habits and developing new ones (Adriaanse et al., 2010; Osbaldiston and Schott, 2012). However, the effect of implementation intentions to reinforce or strengthen an already existing habit might be much smaller and could explain the lack of effect of implementation intentions on cycling to work.

Focusing on a superordinate goal in addition to the subordinate goal also did not show any effect on cycling to work. Based on a goal theoretical perspective, we expected that adding a superordinate goal would foster cycling to work over time as well as generate positive spillover effects across socio-spatial contexts (cycling in leisure time) and across different behavioral domains (exercising and eating). Compared to action steps and implementation intentions, very little research has dealt with the idea that focusing on superordinate goals could maintain the motivation to work toward a goal. To our knowledge, only one study has empirically tested the effect of focusing on superordinate goals when faced with repeated goal-relevant decisions (Fishbach et al., 2006). Thereby, four studies revealed a consistent pattern showing that activating a superordinate goal increased the tendency to act goal-consistent; that is, to make two decisions that both contribute to achieving the shared superordinate goal. These results indicate that focusing on a superordinate goal leads to a longer maintenance of the positive intervention effect, which is not consistent with our results. Importantly, though, whereas Fishbach's study was conducted in a laboratory setting, our study was a large field study. As such, the present findings complement previous research and show the need for further research highlighting possible mechanisms that could lead to the expected effect in a laboratory setting but not in a field study.

Furthermore, adding a focus on a superordinate goal did not influence spillover effects across behaviors. This result also does not support earlier results from similar streams of research, such as research on the effect of social identity on spillover effects. In the environmental domain for example, focusing on or highlighting a pro-environmental identity increases the likelihood of acting in a pro-environmental way and fosters positive spillover effects across different pro-environmental behaviors (Cornelissen et al., 2013; Van der Werff et al., 2014). In the present study, participants who formulated a superordinate goal were asked to think about why cycling to work is important to them and to derive a personal goal starting with “I want to be a person who…,” which highlights the proximity and conceptual similarity of a superordinate goal and social identities (Oyserman and James, 2011; Van der Werff et al., 2014) and would suggest a positive effect of superordinate goals on spillover effects that was not observed. However, it cannot be ruled out that people in the control condition or in the action step condition may not think of a superordinate goal on their own. Goals at different hierarchical levels are associated with each other (Kruglanski et al., 2002). Depending on the association strength, the activation of a subordinate goal can activate an associated superordinate goal. By thinking about the subordinate goal of cycling to work, a connected superordinate may become accessible, without deliberately undergoing a goal manipulation and explicitly activating it. This assumption is further corroborated by a more recent stream of research that states that goals can guide behavior outside of a person's awareness (e.g., Custers et al., 2012). Contextual stimuli such as priming are shown to activate goals unconsciously and guide behavior (Aarts and Dijksterhuis, 2000; Fishbach et al., 2006). Thus, cycling to work or reporting one's cycling effort could unconsciously activate related superordinate goals. The impossibility of experimentally excluding the activation of superordinate goals in the control condition or action step condition may be one reason why no differences between the four conditions on cycling to work and possible spillover effects could be observed.

The lack of the expected spillover effects over time and across behaviors through the goal formulation—action steps, superordinate goals and the combination of them—could further indicate that the present experimental design is only partially suitable for demonstrating the effects of the goal manipulation. First, no negative spillover effects and even positive spillover effects in some behaviors were observed across all experimental groups. This shows that the original campaign has already succeeded in bringing about a positive change in behavior without any additional interventions. While these results shed a good light on the campaign, however, it is a difficult starting point for identifying possible effects of additional intervention groups, which are expected to prevent negative spillover effects and foster positive spillover effects. Second, the goal formulation might have been too weak. The bike-to-work campaign is well-known in Switzerland and the goal of the campaign—to cycle to work at least half of the working days—is in the foreground of the campaign.^2^ It can be hypothesized that an additional superordinate goal or action steps might therefore have little influence in the context of the existing campaign. This assumption is supported by the self-perception theory (Bem, 1972), according to which people infer attitudes from observing their own behavior which then affects their behavior. Participants of the bike-to-work campaign were advised to report their cycling every day during the campaign. This means that the participants considered their cycling behavior on a daily basis. According to the self-perception theory, this promotes cycling behavior independent of the goal manipulation, which could lead to a suppression of the effect of the goal manipulation and thus explain the lack thereof. Finally, it cannot be ruled out that different processes influence the effect on cycling to work and on related behaviors, with different goals triggering different processes (Höchli et al., 2018). For example, subordinate goals may increase self-efficacy which fosters goal pursuit (Bandura, 1997) but run the risk of decreased motivation after achieving a first subordinate goals (Amir and Ariely, 2008), while superordinate goals may increase commitment (Boudrenghien et al., 2013) but may be too vague to be motivating in the moment (Locke and Latham, 2002). It is possible that these processes contradict each other and cancel each other out, and therefore no direct effect of the goal manipulation is visible.

### Limitations

This study has a number of limitations that should be addressed. First, the sample of the study might be biased due to self-selection. Voluntary participation in the bike-to-work campaign already indicates an affinity for cycling compared to the total population. The willingness of the participants to participate in the present study, in addition to taking part in the bike-to-work campaign, results in a sample with highly motivated participants who likely show higher commitment and willingness to cycle to work compared to the other participants in the bike-to-work campaign who did not take part in the present study, and to the general population. However, in this study, it was not possible to compare commitment or behavior to a control group that did not participate in the campaign, as the sample consists exclusively of participants in the bike-to-work campaign. To assess the effect of the campaign more comprehensively, it would be necessary to both (1) look at within-person variance comparing the frequency of cycling to work of a person to his or her baseline level and (2) compared it to a control group not taking part in the campaign.

A second limitation of this study is that self-reporting behaviors leads to several known errors and biases, such as erroneous beliefs about one's behavior or social desirability bias (e.g., Chao and Lam, 2011; Kormos and Gifford, 2014). This shows the need to replicate the results in additional studies that are not based on self-reports. In addition, several longitudinal measurements (the self-reported frequency of cycling to work, cycling in leisure time, and exercising) in this study consisted of single item indicators (frequency of activity per week). It is generally accepted that, in many cases, short measurement instruments are inferior to multi-item measurement instruments, especially as there is no easy statistical way to determine (and report on) their reliability (Diamantopoulos et al., 2012; Postmes et al., 2013). Nevertheless, in this study we deliberately opted for single item measurements for the longitudinal frequency measurements. First, we made this decision for pragmatic reasons: Due to the high number of repeated measurements in this study, we have kept the number of questions as low as possible in order to keep the participant effort at an acceptable level throughout the study (Robins et al., 2001). Secondly, we also opted for single item measurements from a conceptual point of view: Single item measures and short scales can achieve a satisfactory level of reliability when they evaluate homogeneous and clearly defined concepts (Loo and Kelts, 1998; Postmes et al., 2013). The measurement of the frequency of the performance of an activity in a given limited time period seems to be sufficiently homogeneous to be operationalized with a single element. The use of single item measures is further supported by encouraging results from recent research that investigated the comparative reliability and validity of individual items and multi-item measures (Gogol et al., 2014). Having said this, we encourage further research into the behavior of interest using reliable and valid multi-item measurements to identify and complement any weaknesses in the measurement. When undergoing the goal manipulation, the participants of this study formulated their own superordinate goals; this could be seen as a third limitation because it does not allow control over the exact content and behavioral context of the goals. According to the goal systems theory, a superordinate goal is interconnected with several distinct behaviors and vice versa: a behavior can be interconnected with multiple superordinate goals (Kruglanski et al., 2002). Cycling to work, for example, could be connected to the superordinate goal of living a healthy life, but could also be connected to an environmental goal (e.g., leading an environmentally friendly life) or social goal (e.g., being a person who cultivates social contacts). For this reason, it is difficult to make clear predictions as to what extent different behaviors or subordinate goals are related to each other and thus between which behaviors spillover effects are most likely to be expected. When a person focuses on a superordinate goal in the health domain, a spillover effect on healthy eating requires a different interpretation than when a person focuses on a superordinate goal in the environmental domain. In order to avoid this uncertainty, it would be possible to avoid individual formulations of superordinate goals by the participants by setting the same superordinate goal for all participants in the design of the study. But we decided against this course of action due to the personal nature of superordinate goals; these goals describes who a person is trying to be and thus is a central aspect of a person's identity (e.g., Emmons, 1989, 2005; Carver and Scheier, 2001). And as such, it is highly unlikely that a superordinate goal imposed by the intervention design would meet these criteria for all participants.

Finally, no special attention was paid to seasonal effects on the study even though it is colder, rainier, and snowier in Switzerland during the winter. That said, this seasonal change occurs across Switzerland during the winter, and weather and road conditions varied in a similar way for all participants. This is clearly visible in that the entire sample, regardless of the condition, cycled to work significantly less frequently in winter than they did in the baseline measurement in spring. Because data from the different experimental conditions were examined in parallel, it is unlikely that the seasonal variations differentially affected our central research questions. However, when it comes to investigating the main reasons and obstacles which encourage or hinder cycling, weather and seasonal effects as well as conditions for adapting bicycle use, such as road conditions, the presence of cycle paths, distance to the workplace or elevation of terrain, must certainly be considered. Furthermore, in order to investigate the influence of different goal formulations on behavior over time, it would be interesting to observe how cycling behavior develops in the spring and summer following the study. More specifically, it would be interesting to investigate whether the goal manipulation affects the time, extent and intensity that participants start cycling after a winter break.

### Future Research

While the present study sheds light on the effect of interventions in the field over time and across behaviors, most research on spillover effects is still based on correlational studies or laboratory studies with small sample sizes. This makes it difficult to draw causal inferences regarding the effect of an intervention over time and across different behaviors and thus to derive relevant implications for the design of environmental policy. We therefore encourage further experimental field studies (e.g., randomized controlled trials) to achieve a comprehensive understanding of the net effect of an intervention in the field after accounting for possible spillover effects.

The observed positive spillover effects on some behaviors, but not on others, lead to the same conclusion as the inconsistent results on the direction and size of spillover effects from earlier research: In order to understand spillover effects, it is indispensable to examine processes and boundary conditions regarding the effects studied. This concerns both the behavior targeted by an intervention and the behaviors to which a change in the targeted behavior could spill over. More research is needed to understand why spillover effects are more or less likely to occur across some behaviors than others, and to understand the types of behaviors that may be valuable targets for policy interventions after accounting for spillover effects (Dietz et al., 2009; Truelove et al., 2014). The similarity between behaviors and the effort and cost necessary to perform the behavior, or the interconnection with an underlying superordinate goal that relates different behaviors to each other, are promising starting points to shed light on this matter.

Furthermore, our results show that participants with a higher level of individual means of cycling to work showed a slightly larger spillover effect on cycling in leisure time and on exercising than participants with a lower level. This suggests that the existence and size of spillover effects may depend on the frequency or intensity of the targeted behavior prior to intervention. We suggest further research that looks at different levels of expertise, frequency of performance or existing habits regarding the behavior targeted by the intervention. Since many large-scale interventions, such as the bike-to-work campaign, are aimed at a wide range of participants with different starting situations, we expect such insights to be of great practical relevance for policy makers and intervention designers.

Finally, this study shows some evidence that focusing on a superordinate goal in addition to a subordinate goal can increase the positive intervention effect. This suggests that, despite the lack of a clear positive effect in the present studies, a goal theoretical perspective could be a valuable approach to increasing the effectiveness of future interventions. Due to several limitations of the present study—for example, that the control group also participated in the campaign, and that the goal manipulation was carried out within the framework of a campaign with a prevailing and widely known campaign goal—we recommend further experimental studies that highlight the role of superordinate goals and action steps in interventions.

## Conclusion

The present experimental field study reduces the concern that an intervention focusing on a specific behavior over a limited period of time (i.e., a subordinate goal) gives rise to negative spillover effects over time and across behaviors that could nullify or even reverse the intended intervention effect. In addition, the study shows that positive spillover over time and across behaviors is possible, but does not occur consistently, indicating that several additional factors such as the similarity or cost of a behavior or the pre-intervention behavior also affect the presence and size of spillover effects. Although the observed positive spillover effects over time and across behavior cannot be traced back to the goal manipulation, the results give first indications that an additional focus on a superordinate goal can reinforce the intervention effect.

The results show the need for further experimental field research to shed light on the boundary conditions and processes by which positive spillover effects occur, and on the role of a goal theoretical perspective to increase the effectiveness of behavioral change interventions.

## Data Availability

The dataset and R code for this study can be found in the open science framework: https://osf.io/rx9bu/?view_only=e0daaebafb5d44418891216488b3e446.

## Ethics Statement

This study was carried out as part of a larger research project in accordance with the recommendations of the Federal Act on Research involving Human Beings (Human Research Act, HRA) of the Swiss Confederation. The research project was approved by the ethics committee of the canton of Bern, member of the Swiss Ethics Committee on research involving humans.

## Author Contributions

BH, AB, and CM jointly developed the ideas in the paper. BH collected the data. RA merged and prepared the data set. BH and RA analyzed the data. BH wrote the paper. AB and CM read the paper and provided feedback on several drafts of the paper.

### Conflict of Interest Statement

The authors declare that the research was conducted in the absence of any commercial or financial relationships that could be construed as a potential conflict of interest.

## Footnotes

^1^Pseudo-*R*^2^ = [(unrestricted error – restricted error)/unrestricted error] (Raudenbush and Bryk, 2002).

^2^https://www.biketowork.ch/en/
