# Supplementary material for: Using a Goal Theoretical Perspective to Reduce Negative and Promote Positive Spillover After a Bike-to-Work Campaign
Source: Front Psychol. 2019 Mar 6;10:433. doi: 10.3389/fpsyg.2019.00433 (PMC6414797; doi:10.3389/fpsyg.2019.00433)
Supplement: Supplementary file 1 [file Data_Sheet_1.pdf]

Supplementary Material

Using a goal theoretical perspective to reduce negative and promote positive spillover after a bike-to-work campaign

Bettina Höchli*, Adrian Brügger, Roman Abegglen, Claude Messner

*** Correspondence:** Corresponding Author: Bettina.hoechli@imu.unibe.ch

Supplementary Model 1.

Multivariate multilevel model: Effects of the Bike-to-Work Campaign Over Time

This multivariate multilevel model captures the mean difference in the frequency of cycling to work between fixed occasions. To model the differences between the means at the different time points and the mean at baseline we included five dummy variables, each of which contrasts one of the five measurements after the campaign with the baseline measurement before the campaign (Lischetzke et al., 2015). Thus, we included six measures per participant (n = 1269) for a total of 7614 data points.

The intercept, π_{0i}, represents individuals baseline measure of cycling to work before the intervention takes place. We did allow for variance regarding the intercept. The mean difference of cycling to work between two time points is captured by the individual difference scores (π_{1i}–π_{5i}) which depicts the non-reference score minus the reference score of cycling to work of an individual. The difference between two time points is significantly different from zero if the test of the level 2 fixed effects is significant (β_{00}–β_{50}, the mean difference between two time points across individuals). In order to assess the effect of the campaign across all participants, we did not allow for variance in the slope (no error term r_{1i}–r_{5i}) which means that the slope is the same for all participants and the individuals' difference scores equal the mean difference score).

Model 1: Multivariate multilevel model for within-subjects pre- and /post-design with six fixed occasions.

Level 1 (measures): Cycling to work$_{ti}$ = π_{0i} + π_1Post + π_{2i}Follow-up 1 + π_{3i} + Follow-up 2 + π_{4i}Follow-up 3 + π_{5i}Follow-up 4

Level 2 (persons):

$\pi_{0i} = \beta_{00} + r_{oi}$

$\pi_{1i} = \beta_{10}$

$\pi_{2i} = \beta_{20}$

$\pi_{3i} = \beta_{30}$

$\pi_{4i} = \beta_{40}$

$\pi_{5i} = \beta_{50}$

Supplementary Model 2.

Multilevel growth model: Effects of goal type on cycling to work

In order to make statements about how cycling to work will develop after the end of the campaign, model 1 was slightly adapted. The adapted model, model 2, included the baseline measurement of cycling to work as a covariate. The first level of analysis is again at the repeated-measures level—that is, respondents' reported longitudinal measures—but with five measures per participants for a total of 6345 data points; time is set to zero at the time of the end questionnaire (end questionnaire = time 0), to 1 at the time of the follow-up measurement 1 month after the end of the campaign, to 2 at the time of the follow-up measurement 2 months after the campaign, to 3 at the time of the follow-up measurement 3 months after the campaign, and to 7 at the time of the follow-up questionnaire in winter, 7 months after the end of the campaign. The second level of analysis is again at the level of the individual respondent, with $n = 1269$.

We specified and estimated a linear growth model for cycling to work that allowed each participant to have her own initial level of cycling to work (= time point 0 at the end of the campaign) and rate of change in cycling to work. Model comparisons via deviance tests (e.g., Snijders and Bosker, 2012) showed that the random effects had a variance that was significantly larger than zero. The model included the maximal random effects structure justified by the data.

To assess the effect of the goal manipulation on cycling to work over time, cycling to work is the first-level outcome variable and goal type is a second-level, or between-individuals, predictor. Because participants were randomized to treatment and control conditions, we hypothesized no group differences in average baseline measures of cycling to work and included it as a second-level covariate, centered at the grand mean (henceforth denoted with the suffix ".cgm", see Enders and Tofighi, 2007).

Model 2: Multilevel growth model examining the effect of goal type on cycling to work

Level 1 (measures): $\text{Cycling to work}_{ti} = \pi_{0i} + \pi_{1i} * \text{time} + e_{ti}$

Level 2 (persons): $\pi_{0i} = \beta_{00} + \beta_{01} * \text{baseline cycling.cgm} + \beta_{02} * \text{goal type} + r_{oi}$

$\pi_{1i} = \beta_{10} + \beta_{11} * \text{goal type} + r_{1i}$

If there is substantial within-person variability, a multilevel analysis is essential to answering the research question. In order to examine this prerequisite for the present analysis, we first estimated unconditional means models to partition the variance in cycling to work across both levels of analysis and then calculated the intraclass correlation (ICC, proportion of variance that is due to the person level). An ICC of .53 for cycling to work demonstrated substantial within-person variability.

Supplementary Models 3-6.

Spillover effects of the campaign across socio-spatial contexts and across behavioral domains

To assess the effects of cycling to work for four possible spillover behaviors, spillover behavior is taken to be a first-level outcome variable while cycling to work is a first-level predictor variable. In the models described below, the first-level predictor (cycling to work) change is of primary substantive interest. However, without any centering or with only grand-mean centering of a first-level predictor, the slope contains both within- and between-person variation, resulting in a mix of the Level 1 and Level 2 association between the independent and the dependent variables (Enders and Tofighi, 2007). To get a "pure" estimate of the within-person effect, cycling to work was group-mean centered (i.e., centered at the individuals' mean, see Enders and Tofighi, 2007). For simplicity, the person mean-centered variables are henceforth denoted with the suffix ".cwc" (centered within clusters, see Enders and Tofighi, 2007; Raudenbush and Bryk, 2002). As group-mean centering of the level 1 variables removes between-person information, we additionally averaged cycling to work within each individual across all measurement points. We entered these person-means as a second-level predictor in the analysis, in order to test whether cycling to work has a differential effect between persons than within persons on other behaviors (Enders and Tofighi, 2007). Furthermore, we included the cross-level interaction between the first-level predictor *cycling to work* and the second-level predictor *person-means of cycling to work* in order to test for a moderation effect, such that the association between cycling to work and cycling in leisure time is stronger for people who cycle to work more often. Baseline measures of the respective behavior and of cycling to work are included as second-level covariates. In the models below, all second level predictors are centered at the grand mean; for simplicity, the grand mean-centered variables are henceforth denoted with the suffix ".cgm" (Enders and Tofighi, 2007).

Models 3-6: Multilevel models examining the relation between cycling to work and four possible spillover behaviors.

Level 1 (measures): possible spillover $\text{behavior}_{ti} = \pi_{0i} + \pi_{1i}$ * cycling to work.cwc + e_{ti}

Level 2 (persons): $\pi_{0i} = \beta_{00} + \beta_{01}$*baseline cycling.cgm + β_{02} *baseline possible spillover behavior.cgm + β_{03} *person means cycling to work.cgm + r_{oi}

$\pi_{1i} = \beta_{10} + \beta_{11}$ * person means cycling to work.cgm + r_{1i}

We included random effects for the intercept terms and for the slope terms in all models. Model comparisons via deviance tests (e.g., Snijders and Bosker, 2012) showed that all the random effects had a variance that was significantly larger than zero.

An ICC of .61 for cycling in leisure time, .59 for exercising, .71 for eating fruits and vegetables and .62 for eating sweets and snacks demonstrated substantial within-person variability, suggesting that a multilevel analysis is essential to address the research question.

Supplementary Models 7-10.

Spillover effects of the goal-type manipulation across socio-spatial contexts and across behavioral domains

Models 7–10 are based on model 2 with the exception that the spillover behavior replaced cycling to work as the dependent variable, and the baseline of the respective spillover behavior replaced the baseline of cycling to work.

Models 7-10: Multilevel growth model examining the effect of goal type on cycling to work

Level 1 (measures): $\text{possible spillover behavior}_{ti} = \pi_{0i} + \pi_{1i} * \text{time} + e_{ti}$

Level 2 (persons): $\pi_{0i} = \beta_{00} + \beta_{01} * \text{baseline possible spillover behavior.cgm} +$

$\beta_{02} * \text{goal type} + r_{oi}$

$\pi_{1i} = \beta_{10} + \beta_{11} * \text{goal type} + r_{1i}$

Model comparisons via deviance tests (e.g., Snijders and Bosker, 2012) showed that all the random effects had a variance that was significantly larger than zero. Thus, the models 7–10 contain both fixed effects and all possible random effects. An ICC of 0.47 for cycling in leisure time, 0.48 for exercising, 0.62 for eating fruits and vegetables and 0.49 for eating sweets and snacks demonstrated substantial within-person variability, suggesting that a multilevel analysis is essential to address the research question.
(Dietz et al., 2009)

References

Dietz, T., Gardner, G. T., Gilligan, J., Stern, P. C., and Vandenbergh, M. P. (2009). Household actions can provide a behavioral wedge to rapidly reduce US carbon emissions. *Proc. Natl. Acad. Sci.* 106, 18452–18456. doi:10.1073/pnas.0908738106.

Enders, C. K., and Tofighi, D. (2007). Centering predictor variables in cross-sectional multilevel models: a new look at an old issue. *Psychol. Methods* 12, 121–138. doi:10.1037/1082-989X.12.2.121.

Lischetzke, T., Reis, D., and Arndt, C. (2015). Data-analytic strategies for examining the effectiveness of daily interventions. *J. Occup. Organ. Psychol.* 88, 587–622. doi:10.1111/joop.12104.

Raudenbush, S. W., and Bryk, A. S. (2002). *Hierarchical linear models: Applications and data analysis methods*. Thousand Oaks, CA, US: SAGE Publications.

Snijders, T., A. B., and Bosker, R. J. (2012). *Multilevel Analysis: An Introduction to Basic and Advanced Multilevel Modeling*. 2nd edition. London: SAGE Publications.
